# Contribution of tissue oximetry to the assessment of renal dysfunction in abdominal compartment syndrome: an experimental canine study

**DOI:** 10.3389/fsurg.2026.1775062

**Published:** 2026-05-21

**Authors:** Vaia Nalbanti, Stavros Kalfadis, Orestis Ioannidis, Antonia Aikaterini Bourtzinakou, Elissavet Anestiadou, Savvas Symeonidis, Stefanos Bitsianis, Efstathios Kotidis, Konstantinos Zapsalis, Manousos-George Pramateftakis, Ioannis Mantzoros, Stamatios Aggelopoulos

**Affiliations:** Fourth Department of Surgery, Faculty of Health Sciences, Medical School, Aristotle University of Thessaloniki, General Hospital “George Papanikolaou,” Thessaloniki, Greece

**Keywords:** abdominal compartment syndrome, animal model, intra-abdominal hypertension, renal dysfunction, tissue oximetry

## Abstract

**Background:**

Abdominal compartment syndrome (ACS) is a life-threatening condition caused by sustained intra-abdominal hypertension (IAH) leading to multiorgan dysfunction. Renal impairment is a particularly severe consequence, but the underlying mechanisms remain incompletely defined. Impaired tissue oxygenation has been proposed as a key factor, yet studies assessing renal cortical oxygenation during elevated intra-abdominal pressure (IAP) are scarce. This experimental study aimed to evaluate the effects of IAP elevation on renal tissue oxygenation, as well as respiratory and hemodynamic parameters, in a canine model.

**Methods:**

Eight adult dogs (10–20 kg) underwent general anesthesia, mechanical ventilation, and invasive monitoring. Controlled IAP elevation was induced via intraperitoneal CO₂ insufflation. A Clark-type electrode was inserted into the renal cortex to continuously measure renal cortical partial tissue oxygen tension (ptiO₂). Measurements were recorded at baseline, at IAP levels of IAP 15 mmHg and IAP 30 mmHg, and after decompression. Respiratory mechanics, arterial blood gases, hemodynamic variables, oxygen transport variables, and urine output were simultaneously assessed.

**Results:**

Increasing IAP significantly decreased renal ptiO₂ (30% reduction at 15 mmHg, 49% at 30 mmHg; *p* < 0.05). Urine output also declined progressively, with incomplete recovery after decompression. PaO₂ decreased significantly, whereas PaCO₂ showed no statistically significant changes across experimental phases. Lung compliance deteriorated with increasing IAP. Hemodynamically, heart rate, mean arterial pressure, pulmonary capillary wedge pressure, and central venous pressure increased significantly, whereas cardiac output and oxygen delivery remained stable due to fluid support. Following decompression, most parameters improved; however, renal ptiO₂ and diuresis did not fully normalize.

**Conclusion:**

To our knowledge, this represents one of the first direct experimental demonstrations of renal cortical hypoxia under IAH using tissue oximetry, underscoring the role of local oxygen dynamics in pressure-induced renal dysfunction. IAH induces marked renal cortical hypoxia and reduced diuresis, independent of systemic hemodynamic stability. These findings suggest that renal dysfunction in ACS may result from intrarenal blood flow redistribution and parenchymal compression. Tissue oximetry provides valuable real-time insights and may support earlier detection of renal compromise in ACS.

## 1 Introduction

Abdominal compartment syndrome (ACS) is a serious and potentially life-threatening condition characterized by new-onset organ dysfunction due to sustained intra-abdominal pressure (IAP) exceeding 20 mmHg, as defined by the World Society of the Abdominal Compartment Syndrome (WSACS) (1). First described in the 19th century by Marey (1863) and Burt (1870) (2), the clinical relevance of elevated IAP is now well recognized across various surgical and critical care settings, including ascites (3), sepsis, pancreatitis, intestinal obstruction (4), and particularly severe abdominal trauma (5).

Intra-abdominal hypertension (IAH) is commonly encountered among critically ill patients, with reported prevalence rates ranging from 50% to 60% in mixed ICU populations, while ACS develops in approximately 5%–8% of high-risk populations (6). Importantly, ACS is associated with increased mortality rates, ranging between 40% and 70%, depending on the underlying etiology and timing of decompression (7). Renal dysfunction is among the earliest and most frequent manifestations of increased IAP, and IAH-associated acute kidney injury (AKI) has been strongly linked to increased morbidity, prolonged intensive care stay, and mortality exceeding 50% (8).

According to WSACS guidelines (9), intra-abdominal hypertension (IAH) is stratified into four grades based on IAP levels:

- Grade I: 12–15 mmHg
- Grade II: 16–20 mmHg
- Grade III: 21–25 mmHg
- Grade IV: >25 mmHg

Despite established classifications, clinical observations reveal significant interindividual variability in physiological responses to IAH. Not all patients exhibit the same degree of physiological derangement at equivalent IAP levels (10), and it remains unclear why some progress to ACS while others do not (11). IAH exerts extensive systemic effects due to the transmission of elevated pressure from the abdominal cavity to adjacent organ systems. Intra-abdominal hypertension (IAH) exerts widespread systemic effects through transmission of elevated pressure from the abdominal cavity to adjacent organ systems. Hemodynamically, IAH can lead to a reduction in venous return by compressing the inferior vena cava, thereby diminishing preload and cardiac output (CO) (12, 13). This reduction in forward flow is often accompanied by a reflex increase in systemic vascular resistance, as part of a compensatory response to maintain arterial pressure and organ perfusion (14).

Respiratory compromise occurs as elevated IAP displaces the diaphragm cephalad, reducing thoracic compliance, increasing peak airway pressures, and decreasing functional residual capacity. These changes predispose to hypoxemia, hypercapnia, and impaired ventilation–perfusion matching, particularly in mechanically ventilated patients (15).

Renal function is particularly vulnerable to the effects of IAH. Although numerous experimental and clinical studies have explored IAH-related renal impairment, its precise mechanisms remain incompletely elucidated (16–18). Increased pressure within the abdominal cavity can lead to direct renal parenchymal compression, elevated renal vein pressure, and a reduction in renal arterial inflow, collectively resulting in decreased glomerular filtration rate (GFR) (19). The renal medulla—which normally operates near hypoxic thresholds—may become critically ischemic under conditions of intra-abdominal hypertension, further impairing nephron function. Clinically, these pathophysiological alterations manifest as oliguria or anuria, even in the absence of overt systemic hypotension (20, 21). In severe cases, prolonged exposure to elevated IAP can precipitate AKI through both prerenal and intrinsic mechanisms (22).

Importantly, both surgical interventions (e.g., decompressive laparotomy) and non-surgical measures (e.g., nasogastric decompression, optimization of sedation, and diuretic therapy) for reducing IAP can result in prompt reversal of these physiological derangements. Restoration of abdominal wall compliance and reduction of intra-abdominal volume can promptly improve systemic hemodynamics, gas exchange, and renal perfusion. This highlights the dynamic and reversible nature of IAH-induced organ dysfunction, particularly when timely recognition and intervention are achieved (23)*.*

Tissue oxygenation is a fundamental determinant of organ viability and function, governed by the delicate balance between oxygen delivery—dependent on perfusion and hemoglobin content—and oxygen consumption at the cellular level (24). Disruption of this balance, particularly in metabolically active organs such as the kidney, can precipitate cellular hypoxia and subsequent organ dysfunction. In this context, tissue oximetry represents a novel investigative modality that enables continuous, real-time assessment of local oxygen tension within the parenchyma via specialized intratissue electrodes (25). Although this technique has been employed in studies of cerebral and muscular oxygenation (26, 27), its application to renal tissue oxygenation under conditions of intra-abdominal hypertension has been limited. Notably, Kalfadis et al. (28) previously investigated renal cortical oxygenation in an experimental canine model under the combined presence of abdominal compartment syndrome and sepsis, demonstrating a significant reduction in renal tissue oxygen tension under these complex pathophysiological conditions. While that study provided important insights into renal hypoxia during the coexistence of intra-abdominal hypertension and systemic inflammation, it did not allow isolation of the specific contribution of elevated intra-abdominal pressure itself.

The objective of the present experimental study was to evaluate early alterations in renal function associated with abdominal compartment syndrome through the use of direct tissue oximetry. This technique offers the unique advantage of capturing early and dynamic alterations in renal oxygenation, potentially preceding overt clinical signs of hypoperfusion or ischemia. The present study was designed as a reductionist experimental model to isolate the effects of elevated intra-abdominal pressure on renal oxygenation in the absence of septic or inflammatory confounders. By characterizing changes in renal cortical oxygen tension during graded increases in intra-abdominal pressure under controlled hemodynamic conditions, this study aimed to elucidate pressure-mediated mechanisms contributing to abdominal compartment syndrome-associated acute kidney injury.

## 2 Materials and methods

### 2.1 Experimental animals 

A total of eight (*n* = 8) non-breed adult dogs of both sexes, weighing between 10 and 20 kg, were included in the study. The choice of canines was based on their physiological similarities to humans in terms of hemodynamic, respiratory, and renal functions (29). All procedures were designed to be fully reversible and did not result in long-term adverse outcomes. No animals were euthanized as part of the experiment.

### 2.2 Anesthesia and ventilation

Preanesthetic sedation was achieved with intramuscular xylazine hydrochloride (ROMPUN® 2%, Bayer) at a dose of 1 mL/kg. After 30 min, percutaneous catheterization of the right brachial vein was performed with an 18G angiocatheter (Abocath®-T), followed by intravenous induction of general anesthesia using thiopental sodium (Pentothal®, Abbott) 1.25% at a dose of 5 mg/kg.

Tracheal intubation was performed with a Shiley endotracheal tube, and animals were mechanically ventilated using a Siemens SERVO 900C ventilator. Ventilation parameters were adjusted to maintain PaO₂ > 100 mmHg, PaCO₂ between 30 and 50 mmHg, and arterial pH within the 7.2–7.6 range.

Anesthesia was maintained via continuous infusion of thiopental sodium at 2 mg/kg/h. Analgesia was provided using fentanyl (JANSSEN) at a rate of 15 µg/kg/h, and neuromuscular blockade was achieved with atracurium at 1 mg/kg/h, chosen for its organ-independent metabolism. Ringer's lactate and normal saline were administered intravenously to maintain hemodynamic stability.

Mechanical ventilation was delivered in volume-controlled ventilation mode. Tidal volume (Vt) was set at 10–12 mL/kg, respiratory rate (f) was adjusted between 10 and 16 breaths/min to maintain target PaCO₂ values, and positive end-expiratory pressure was set at 0 cmH₂O throughout the experiment. Ventilator settings were kept constant across experimental phases, with adjustments limited to respiratory rate as needed to maintain arterial blood gas targets.

### 2.3 Instrumentation and monitoring

**Figure 1:**
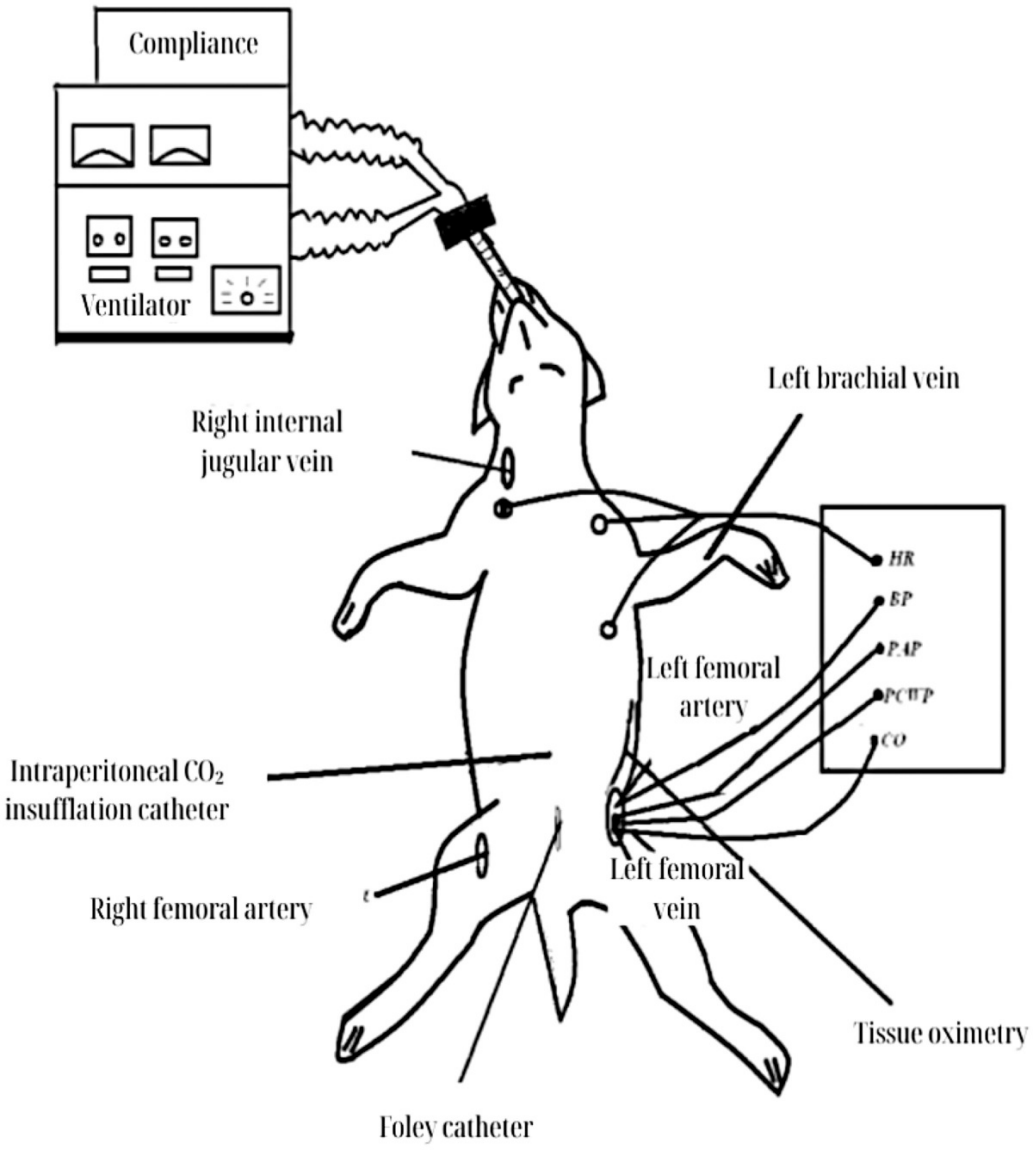
Instrumentation and monitoring layout in the experimental canine model. Diagram showing vascular access (arterial and venous), pulmonary artery catheterization, intraperitoneal CO₂ insufflation for IAP modulation, renal tissue oximetry probe placement, ventilator connection for respiratory monitoring, and urinary catheterization for diuresis assessment.

Pulmonary compliance was continuously monitored using the integrated Lung Mechanics Calculator 940 (Siemens, Erlangen, Germany). Continuous electrocardiographic monitoring, using lead II, was performed with a Datex-Engstrom CS/3 monitor (Datex-Engstrom, Helsinki, Finland). After surgical exposure of the left femoral artery and vein, the following catheters were placed (Figure 1):

- Femoral artery: A 16G angiocatheter (Abocath®-T) was inserted for continuous blood pressure monitoring and arterial blood sampling. Arterial blood gas analysis was performed using the ABL 330 gas analyzer (Radiometer Copenhagen), and hemoglobin concentrations were measured with the Hemoximeter OSM 3.
- Femoral vein: A Swan-Ganz catheter (8F, Opti-Q®, Abbott) was advanced through the inferior vena cava to the pulmonary artery. This allowed measurement of right atrial pressure, pulmonary capillary wedge pressure (PCWP), and continuous cardiac output.
- Peritoneal cavity/peritoneal insufflation system: A catheter with a one-way valve was inserted into the peritoneal cavity for carbon dioxide insufflation to induce controlled increases in IAP, using a SOLOS ENDOSCOPY PAP III insufflator (Solos Endoscopy, Tüttlingen, Germany).

### 2.4 Surgical procedure

A left lateral thoracolumbar incision approximately 10 cm in length was performed to expose the left kidney. A tissue oximetry probe was carefully inserted 3 mm beneath the renal capsule, within the cortex, and secured in place. Tissue oxygenation and temperature were continuously recorded using the Tissutrak monitoring system (Pfizer Biomedical Sensors Ltd., UK).

To induce and regulate intra-abdominal hypertension, a 5-mm trocar (VERSAPORT®, Autosuture) was inserted via a midline subumbilical incision to allow carbon dioxide insufflation and IAP regulation.

### 2.5 Experimental protocol

Following completion of instrumentation, a 30-min stabilization period was observed (*t* = 0). Measurements were subsequently recorded during four predefined experimental phases:

- Phase 1 (Baseline): normal intra-abdominal pressure.
- Phase 2: IAP elevated to 15 mmHg; measurements recorded after a 30-min equilibration period.
- Phase 3: IAP elevated to 30 mmHg; measurements recorded after stabilization period.
- Phase 4 (Decompression): measurements recorded following release of intra-abdominal pressure and restoration of IAP to normal levels.

**Figure 2:**
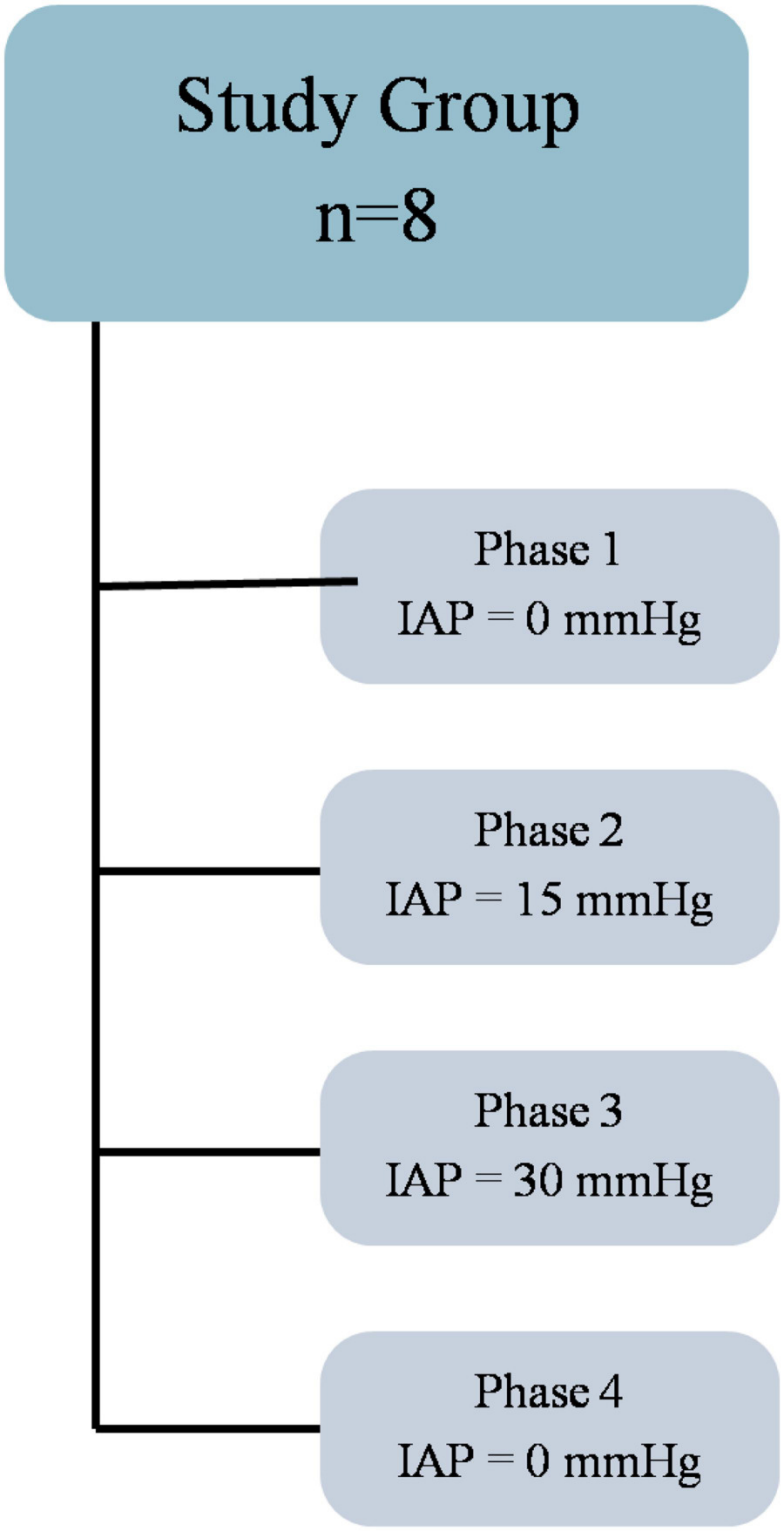
Outline of the study protocol. The four-phase experimental sequence: baseline (IAP 0 mmHg), IAP elevation to 15 and 30 mmHg, followed by abdominal decompression (IAP 0 mmHg). Animals were ventilated with 40% FiO₂ and continuously monitored.

**Figure 3:**
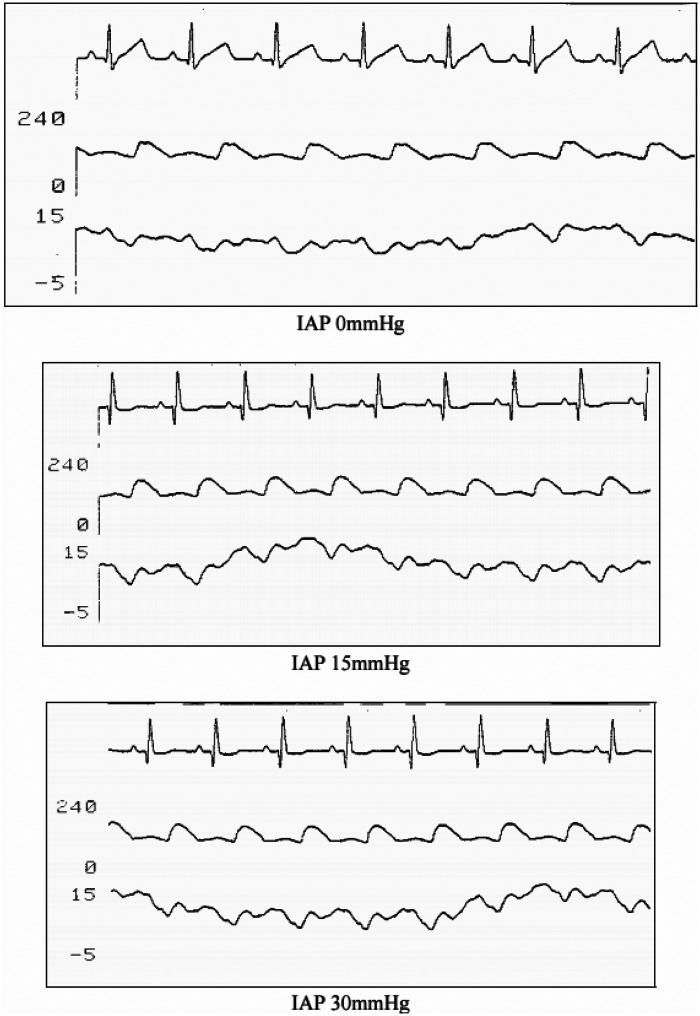
Hemodynamic monitoring under increasing intra-abdominal pressure. Representative parallel recordings of electrocardiography, arterial blood pressure, and central venous pressure obtained at three intra-abdominal pressure (IAP) levels: baseline (IAP 0 mmHg), IAP 15 mmHg, and IAP 30 mmHg. Progressive elevation of IAP is associated with visible alterations in arterial pressure waveform and central venous pressure, while cardiac rhythm remains stable. These tracings illustrate the hemodynamic effects of increasing intra-abdominal pressure during the experimental protocol.

**Figure 4:**
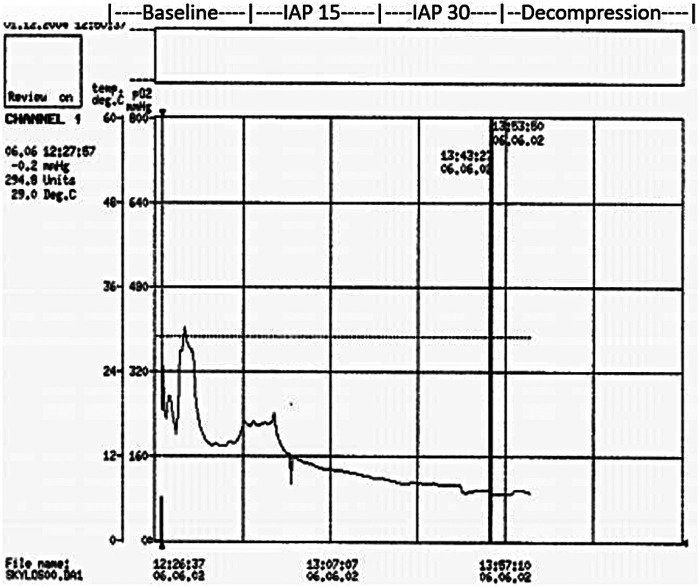
Renal cortical tissue oxygen tension recording. Continuous recording of renal cortical tissue oxygen tension (ptiO₂) obtained using a Clark-type intraparenchymal oxygen electrode in a representative experimental animal. The tracing demonstrates baseline values followed by a progressive decline during graded elevation of intra-abdominal pressure and partial recovery after decompression. The experimental phases (baseline, IAP 15 mmHg, IAP 30 mmHg, and decompression) are indicated along the recording timeline.

Each phase lasted 30 min, during which respiratory, hemodynamic, and renal parameters were recorded (Figure 2). At the end of each phase, simultaneous arterial and venous blood samples were collected for gas analysis, hemoglobin concentration, and electrolyte analysis. Representative raw tracings of hemodynamic parameters and renal tissue oxygen tension are provided to illustrate the physiological response to graded intra-abdominal pressure elevation (Figures 3, 4).

### 2.6 Parameters measured

A comprehensive set of physiological parameters was monitored throughout the study to evaluate the systemic and renal effects of elevated intra-abdominal pressure. These parameters were categorized as follows:

- Respiratory and Gas Exchange Parameters: FiO₂, PaO₂, PaCO₂, pH, base excess (BE), hemoglobin oxygen saturation (Sat), peak airway pressure, and pulmonary compliance.
- Hemodynamic Parameters: heart rate (HR), mean arterial pressure (MAP), mean pulmonary artery pressure (MPAP), PCWP, central venous pressure (CVP), CO, and cardiac index (CI).
- Oxygen Transport and Utilization: oxygen consumption (VO₂), oxygen delivery (DO₂), arteriovenous oxygen difference [D(a–v)], systemic vascular resistance index (SVRI), and pulmonary vascular resistance index (PVRI).
- Renal Parameters: renal tissue oxygen tension (PtiO₂) and diuresis.

### 2.7 Statistical analysis

This experiment included a total of eight (*n* = 8) adult dogs, all of which completed the full four-phase protocol and were included in the final analysis. No animals were excluded from the study and no missing data were recorded for any outcome variable. As all measurements were successfully obtained at each predefined experimental phase, no imputation methods were required. Owing to the within-subject repeated-measures design, each animal served as its own control across all experimental phases.

Data distribution was assessed for normality using the Shapiro–Wilk test. As all experimental phases were assessed sequentially in the same animals, a repeated-measures design was applied. Accordingly, one-way repeated-measures analysis of variance (ANOVA) was used to evaluate overall differences across the four experimental phases (baseline, IAP 15 mmHg, IAP 30 mmHg, and decompression).

The assumption of sphericity was tested using Mauchly's test. When sphericity was violated, degrees of freedom were adjusted using the Greenhouse–Geisser correction (or Huynh–Feldt correction, as appropriate). If the repeated-measures ANOVA demonstrated a statistically significant overall effect, Bonferroni-adjusted *post hoc* pairwise comparisons were performed.

For variables that did not meet normality assumptions, the Friedman test was used, followed by Wilcoxon signed-rank tests with Bonferroni correction for *post hoc* comparisons.

Continuous variables are summarized as mean ± standard deviation in figures and Supplementary Table 1, whereas the Results section provides a structured narrative description of the phase-related changes together with the corresponding statistical comparisons. Statistical significance was defined as *p* < 0.05. All analyses were performed using IBM SPSS Statistics for Windows, Version 26.0 (IBM Corp., Armonk, NY, USA).

The study employed a repeated-measures design, with each animal serving as its own control across all experimental phases. Using the paired renal cortical tissue oxygen tension (ptiO₂) data, within-subject effect sizes (Cohen's dz) were calculated to quantify the magnitude of phase-related changes in the primary outcome.

Relative to baseline, renal cortical ptiO₂ demonstrated a marked reduction at IAP 15 mmHg (≈30%) and a further decrease at IAP 30 mmHg (≈49%), with partial but incomplete recovery following decompression. The corresponding within-subject effect sizes were large (Cohen's dz = 1.09 at IAP 15 mmHg; dz = 1.74 at IAP 30 mmHg; dz = 0.98 for decompression vs. IAP 30 mmHg; and dz = 1.12 for decompression vs. baseline).

Based on these observed effect sizes, the achieved statistical power (two-sided *α* = 0.05) was approximately 0.75 for the comparison between baseline and IAP 15 mmHg and greater than 0.95 for the comparison between baseline and IAP 30 mmHg. These findings indicate that a sample size of eight animals was sufficient to detect physiologically and clinically meaningful within-subject changes in renal cortical oxygenation. The selected sample size is consistent with comparable invasive tissue oximetry studies and adheres to the ethical principle of animal reduction (3Rs) (30).

For graphical data presentation, individual subject trajectories are displayed as thin lines across experimental phases, overlaid with mean ± standard deviation values. This approach was adopted to better illustrate inter- and intrasubject variability over time. For the primary renal outcomes, significant Bonferroni-adjusted *post hoc* pairwise comparisons are additionally displayed within the corresponding figures.

*Post hoc* pairwise comparisons were performed only when the overall ANOVA demonstrated statistical significance, in order to avoid overinterpretation of non-significant findings.

## 3 Results

Detailed descriptive data are presented in the figures and Supplementary Table 1, with the text providing summaries of the direction, magnitude, and statistical significance of the observed changes across experimental phases. Mean ± standard deviation values are provided to facilitate interpretation of the findings.

### 3.1 Respiratory and gas exchange parameters

#### 3.1.1 Fraction of inspired oxygen

The fraction of inspired oxygen (FiO₂) was maintained at 40% throughout all experimental phases. Repeated-measures ANOVA demonstrated no significant effect of experimental phase on FiO₂ (*p* > 0.05). Arterial oxygen tension (PaO₂) remained consistently above 100 mmHg during all phases.

#### 3.1.2 Partial pressure of oxygen in arterial blood (PaO₂)

**Figure 5:**
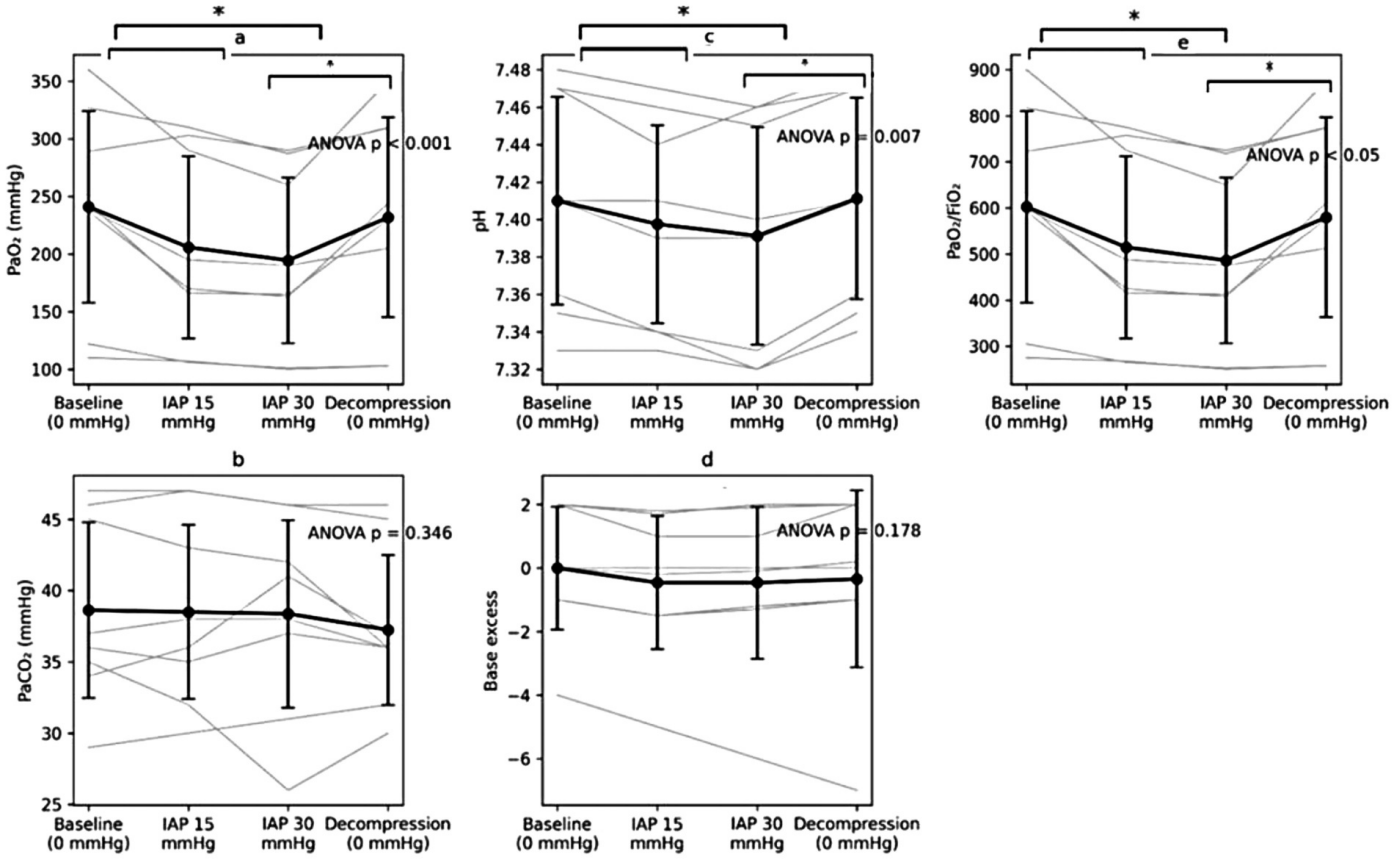
Respiratory blood gas parameters across experimental phases. **(a)** PaO₂ decreased with increasing intra-abdominal pressure (IAP) and improved after decompression. **(b)** PaCO₂ showed no significant changes across experimental phases. **(c)** Arterial pH decreased during IAP elevation and returned toward baseline following decompression. **(d)** Base excess showed no significant changes across experimental phases. **(e)** The PaO₂/FiO₂ ratio demonstrated a similar phase-dependent pattern to PaO₂. Values are presented as mean ± SD, with individual animal trajectories shown as thin lines. The reported *P*-values correspond to the overall repeated-measures ANOVA across all experimental phases. Significant Bonferroni-adjusted *post hoc* pairwise comparisons are indicated within panels **(a)**, **(c)**, and **(e)** only (*p < 0.05, **p < 0.01).

Repeated-measures analysis of variance revealed a significant overall effect of intra-abdominal pressure on PaO₂ (*p* < 0.001). Arterial PaO₂ decreased progressively with increasing intra-abdominal pressure, from 240 ± 85 mmHg at baseline to 205 ± 80 mmHg at 15 mmHg and 195 ± 75 mmHg at 30 mmHg, followed by partial recovery after decompression (230 ± 85 mmHg). *Post hoc* Bonferroni comparisons demonstrated a significant reduction in PaO₂ at 15 mmHg compared with baseline, with a further decrease at 30 mmHg (*p* < 0.05). Following decompression, PaO₂ increased compared with 30 mmHg (*p* < 0.05) but remained lower than baseline values. No significant difference was observed between the two 0-mmHg phases (baseline vs. decompression) (Figure 5a).

Given that the fraction of inspired oxygen was maintained at 0.4 throughout the experiment, the PaO₂/FiO₂ ratio followed a similar pattern, with a decrease at higher intra-abdominal pressure levels and partial recovery after decompression. Importantly, PaO₂/FiO₂ values remained within physiological ranges across all phases, indicating preserved oxygenation despite statistically significant changes.

#### 3.1.3 Partial pressure of carbon dioxide in arterial blood (PaCO₂)

Repeated-measures analysis of variance demonstrated no statistically significant effect of experimental phase on PaCO₂ (*p* = 0.346). Arterial PaCO₂ values remained relatively stable across experimental phases, with mean values of 38 ± 4 mmHg at baseline, 38 ± 5 at 15 mmHg, and 38 ± 7 at 30 mmHg, followed by 37 ± 5 mmHg after decompression. Importantly, PaCO₂ remained within physiological ranges throughout the experiment, indicating preserved carbon dioxide elimination (Figure 5b).

#### 3.1.4 Arterial pH and BE

Repeated-measures analysis of variance identified a significant overall effect of experimental phase on arterial pH (*p* = 0.007). Arterial pH decreased from 7.41 ± 0.05 at baseline to 7.39 ± 0.05 at 15 mmHg and 7.39 ± 0.06 at 30 mmHg, with recovery following decompression (7.41 ± 0.05). *Post hoc* comparisons demonstrated a significant decrease in pH at 15 mmHg compared with baseline. At 30 mmHg, pH remained lower than baseline values, although no additional statistically significant difference was observed compared with the 15 mmHg phase. Following decompression, pH increased compared with 30 mmHg and returned to baseline values (Figure 5c). Importantly, arterial pH values remained within physiological ranges across all experimental phases, indicating preserved acid–base balance despite statistically significant changes.

Similarly, repeated-measures analysis of variance demonstrated no statistically significant effect of experimental phase on base excess (*p* = 0.178). Base excess values remained relatively stable across experimental phases, with mean values of −1.0 ± 2.0 mmol/L at baseline, −1.5 ± 2.2 mmol/L at 15 mmHg, −1.4 ± 2.5 mmol/L at 30 mmHg, and −0.8 ± 2.1 mmol/L after decompression. These changes were not statistically significant, and values remained within physiological ranges throughout the experiment (Figure 5d).

#### 3.1.5 Hemoglobin oxygen saturation (SaO₂)

Repeated-measures ANOVA showed no significant effect of experimental phase on hemoglobin oxygen saturation (*p* > 0.05). SaO₂ remained consistently above 99.4% across all experimental phases.

#### 3.1.6 Peak airway pressure and lung compliance

Repeated-measures ANOVA demonstrated a significant overall effect of intra-abdominal pressure on peak airway pressure and lung compliance (*p* < 0.05 for both). *Post hoc* analysis revealed that peak airway pressure increased significantly at IAP 15 and 30 mmHg compared with baseline and returned to baseline values after decompression.

**Figure 6:**
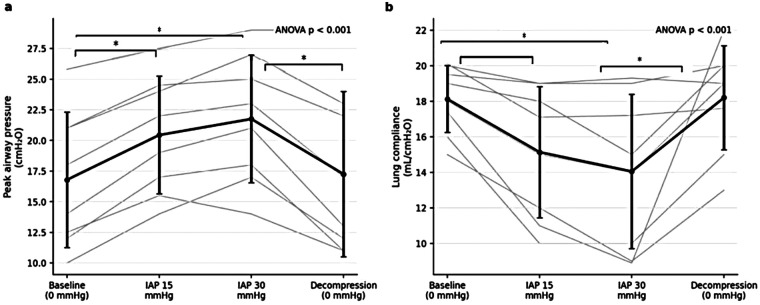
Respiratory mechanics across experimental phases. **(a)** Peak airway pressure increased significantly at IAP 15 and 30 mmHg compared with baseline and returned toward baseline following decompression. **(b)** Lung compliance decreased significantly during IAP elevation (15 and 30 mmHg) and recovered after decompression. Values are presented as mean ± SD, with individual animal trajectories shown as thin lines. The reported *P*-values correspond to the overall repeated-measures ANOVA across all experimental phases. Significant Bonferroni-adjusted *post hoc* pairwise comparisons are indicated within each panel (*p < 0.05, **p < 0.01).

Lung compliance decreased significantly at both IAP 15 and 30 mmHg and recovered following decompression (*p* < 0.05) (Figures 6a, b).

### 3.2 Hemodynamic parameters

#### 3.2.1 Heart rate

**Figure 7:**
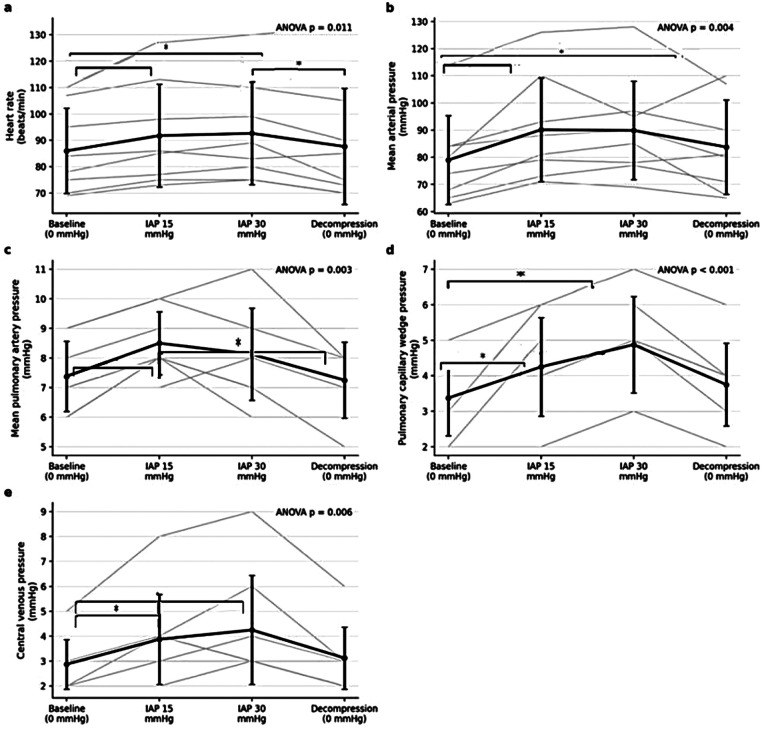
Hemodynamic responses across experimental phases. **(a)** Heart rate increased significantly with rising intra-abdominal pressure (IAP) and returned to baseline following decompression. **(b)** Mean arterial pressure increased at IAP 15 mmHg and remained elevated after decompression, with no significant difference between IAP 15 mmHg and decompression phases. **(c)** Mean pulmonary artery pressure increased at IAP 15 mmHg and returned to baseline following decompression. **(d)** Pulmonary capillary wedge pressure increased at elevated IAP levels and decreased after decompression without full normalization. **(e)** Central venous pressure increased with IAP and decreased following decompression, without a statistically significant difference between baseline and decompression. Values are presented as mean ± SD, with individual animal trajectories shown as thin lines. The reported *P*-values correspond to the overall repeated-measures ANOVA across all experimental phases. Significant Bonferroni-adjusted *post hoc* pairwise comparisons are indicated within each panel (*p < 0.05, **p < 0.01).

Repeated-measures ANOVA demonstrated a significant overall effect of intra-abdominal pressure on heart rate (*p* < 0.05). *Post hoc* Bonferroni analysis revealed a significant increase in HR at IAP 15 mmHg, with a further increase at IAP 30 mmHg compared with baseline (*p* < 0.05). Following decompression, HR decreased significantly and returned to baseline values, with no significant difference between the two 0-mmHg phases (Figure 7a).

#### 3.2.2 Mean arterial pressure

Repeated-measures ANOVA showed a significant phase-dependent effect on MAP (*p* < 0.05). *Post hoc* comparisons demonstrated a significant increase in MAP at IAP 15 mmHg compared with baseline, which persisted after decompression (*p* < 0.05). MAP values during the decompression phase did not differ significantly from those observed at IAP 15 mmHg (Figures 7a, b).

#### 3.2.3 Mean pulmonary artery pressure

A significant overall effect of experimental phase on MPAP was identified by repeated-measures ANOVA (*p* < 0.05). *Post hoc* analysis showed that MPAP increased significantly at IAP 15 mmHg and returned to baseline values following decompression, with no significant difference between baseline and decompression phases (Figure 7c).

#### 3.2.4 Pulmonary capillary wedge pressure

Repeated-measures ANOVA revealed a significant effect of intra-abdominal pressure on PCWP (*p* < 0.05). PCWP increased significantly at both IAP 15 and 30 mmHg compared with baseline. Although PCWP decreased following decompression, values remained elevated relative to baseline, and the difference between baseline and decompression did not reach statistical significance (Figure 7d).

#### 3.2.5 Central venous pressure

Repeated-measures ANOVA demonstrated a significant phase effect on CVP (*p* < 0.05). CVP increased significantly at elevated IAP levels and decreased following decompression, although values did not fully return to baseline. No statistically significant difference was observed between baseline and decompression phases (Figure 7e).

#### 3.2.6 Cardiac output and cardiac Index

Repeated-measures ANOVA showed no significant effect of experimental phase on either cardiac output or cardiac index (*p* > 0.05 for both).

### 3.3 Oxygen transport and utilization

#### 3.3.1 Oxygen consumption (VO₂)

**Figure 8:**
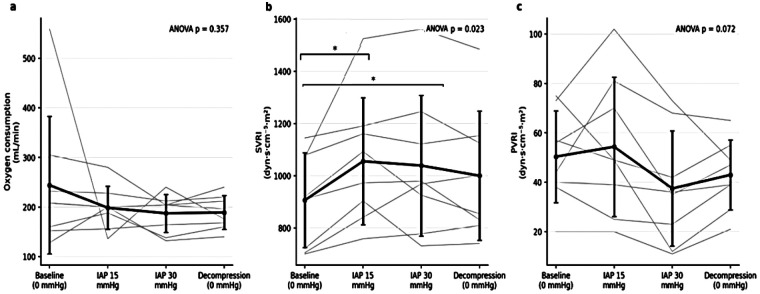
Oxygenation and vascular resistance indices across experimental phases. **(a)** Oxygen consumption (VO₂) showed no significant changes across intra-abdominal pressure (IAP) phases. **(b)** Systemic vascular resistance index (SVRI) increased significantly at IAP 15 mmHg and remained elevated during subsequent phases. **(c)** Pulmonary vascular resistance index (PVRI) showed no statistically significant changes across experimental phases. Values are presented as mean ± SD, with individual animal trajectories shown as thin lines. The reported *P*-values correspond to the overall repeated-measures ANOVA across all experimental phases. Significant Bonferroni-adjusted *post hoc* pairwise comparisons are indicated within panel **(b)** only (*p < 0.05, **p < 0.01).

Repeated-measures ANOVA demonstrated no statistically significant effect of experimental phase on oxygen consumption (*p* > 0.05). Oxygen consumption values remained relatively stable across all experimental phases (Figure 8a).

#### 3.3.2 Oxygen delivery (DO₂) and arteriovenous oxygen difference [D(a–v)]

Repeated-measures ANOVA revealed no statistically significant effect of experimental phase on oxygen delivery (DO₂) (*p* > 0.05). DO₂ values remained relatively stable across all phases, with only minor interindividual variability and no consistent reduction during intraabdominal pressure (IAP) elevation. Similarly, no significant phase-dependent changes were observed in the arteriovenous oxygen difference [D(a–v)O₂] (*p* > 0.05), with values remaining essentially unchanged throughout the experiment. These results indicate the preservation of systemic oxygen transport and extraction despite increasing IAP.

#### 3.3.3 Systemic vascular resistance index

Repeated-measures ANOVA revealed a significant overall effect of experimental phase on SVRI (*p* < 0.05). *Post hoc* analysis showed a significant increase in SVRI at IAP 15 mmHg, which remained elevated compared with baseline throughout subsequent phases, including decompression (Figure 8b).

#### 3.3.4 Pulmonary vascular resistance index

No statistically significant phase-dependent effect was observed for PVRI (*p* = 0.072). PVRI values showed some variability across experimental phases, with a decrease observed at higher intra-abdominal pressure levels and partial recovery following decompression; however, these changes did not reach statistical significance. Accordingly, no *post hoc* pairwise comparisons were performed (Figure 8c).

### 3.4 Renal parameters

#### 3.4.1 Renal tissue oxygen tension (ptiO₂)

**Figure 9.**
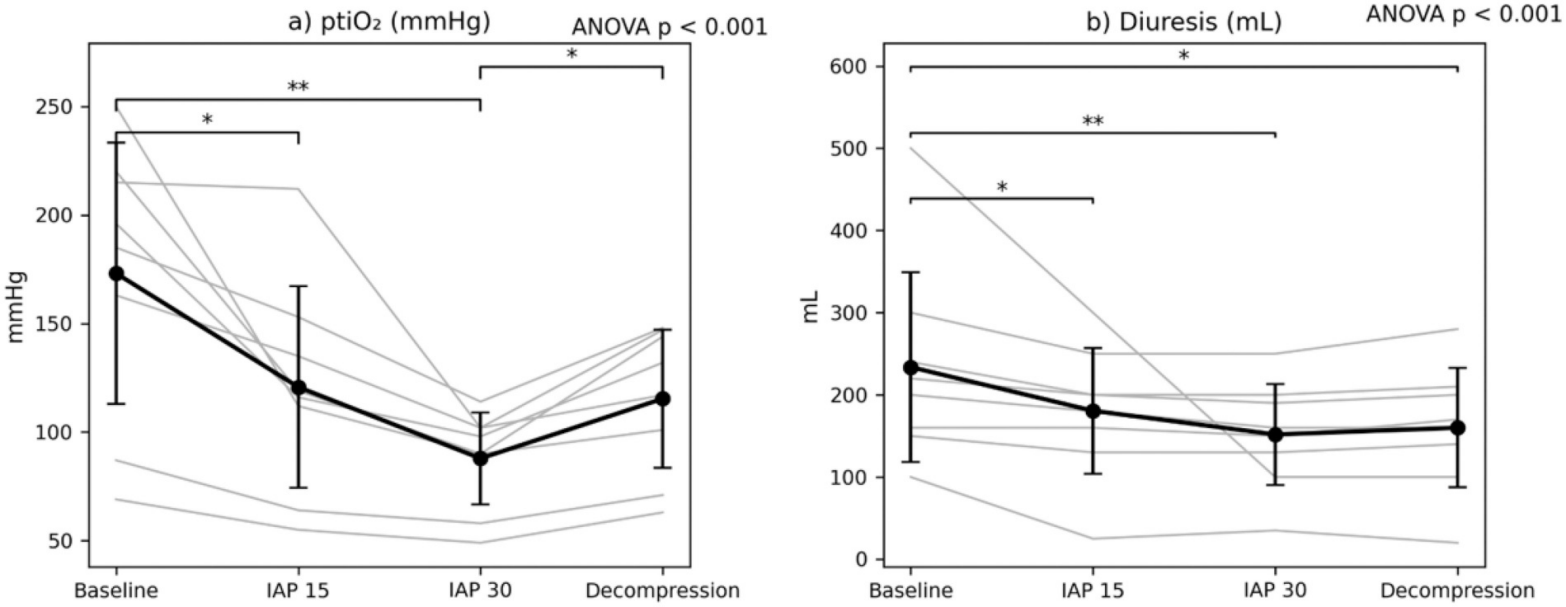
Renal oxygenation and urinary output under varying intra-abdominal pressures (IAP). **(a)** Renal cortical tissue oxygen tension (ptiO₂) decreased progressively with increasing IAP and partially recovered after decompression. **(b)** Diuresis declined significantly during IAP elevation and remained reduced after decompression. Values are presented as mean ± SD, with individual animal trajectories shown as thin lines. The reported *P*-value corresponds to the overall repeated-measures ANOVA across all experimental phases. Significant Bonferroni-adjusted *post hoc* pairwise comparisons are indicated within each panel (*p < 0.05, **p < 0.01).

Repeated-measures ANOVA demonstrated a significant overall effect of intra-abdominal pressure on renal cortical tissue oxygen tension (*p* < 0.05). *Post hoc* Bonferroni analysis revealed significant reductions in ptiO₂ at IAP 15 mmHg and IAP 30 mmHg compared with baseline, as well as a significant increase following decompression compared with IAP 30 mmHg (Figure 9a).

#### 3.4.2 Diuresis

Repeated-measures ANOVA identified a significant phase-dependent reduction in urine output (*p* < 0.05). Diuresis decreased progressively with increasing IAP and remained significantly reduced after decompression, indicating persistent renal functional impairment (Figure 9b).

## 4 Discussion

In recent decades, substantial research has been focused on understanding the physiological effects of IAH, particularly its pathophysiological impact on vital organ systems (31). Among the most clinically significant consequences of sustained elevated IAP is the development of ACS, a condition characterized by progressive and potentially reversible multiorgan dysfunction. Despite increased awareness and advances in monitoring, the pathophysiological mechanisms through which elevated IAP leads to organ failure remain incompletely understood.

Renal dysfunction represents one of the earliest and most consistent manifestations of IAH. Although numerous experimental and clinical studies have investigated this phenomenon, the precise mechanisms underlying IAH-associated renal impairment appear multifactorial and remain only partially elucidated (32). Initial theories that attributed oliguria and anuria to ureteral compression have been invalidated, as ureteral stenting has proven ineffective in preventing renal dysfunction (33). Subsequent studies have demonstrated that increased IAP directly raises renal venous and parenchymal pressures due to direct compression (8). The combination of increased parenchymal pressure, and thus pressure in the proximal tubules, along with reduced renal perfusion was found to decrease the transcapillary pressure gradient across the glomerular membrane, ultimately leading to a significant reduction in GFR (34). Additional evidence supports that elevated IAP causes redistribution of intrarenal blood flow, particularly a shift from the cortex to the medulla, and stimulates the release of antidiuretic hormone (ADH), exacerbating sodium and water retention (35). Furthermore, while reduced cardiac output has traditionally been considered a contributing factor to renal impairment, dysfunction has also been observed in its absence, suggesting other underlying mechanisms (14).

While previous experimental work has demonstrated impaired renal tissue oxygenation in complex models combining intra-abdominal hypertension with systemic inflammation or sepsis, the isolated effects of elevated intra-abdominal pressure on renal oxygenation have not been fully characterized. The present study therefore focused on renal cortical tissue oxygenation under controlled conditions designed to minimize systemic confounders. Our findings demonstrate that graded elevation of intra-abdominal pressure induces a pronounced and progressive reduction in renal cortical tissue oxygen tension, accompanied by a parallel decline in urine output, despite preservation of cardiac output and global oxygen delivery. These results indicate that renal hypoxia develops early during IAH and may occur independently of overt systemic hemodynamic instability. Collectively, the data support the concept that pressure-mediated intrarenal mechanisms play a pivotal role in the pathogenesis of ACS-associated renal dysfunction (9, 28, 36, 37).

The respiratory alterations observed with increasing IAP in the present experiment are in line with well-described physiological responses to abdominal compartment loading. Elevation of the diaphragm and reduced chest wall compliance lead to increased airway pressures, reduced lung compliance, and impaired gas exchange, findings consistently reported in both experimental models and critically ill patients with IAH (37–40). In our canine model, the progressive decline in PaO₂ levels and their subsequent improvement after decompression support the concept that pulmonary alterations during intra-abdominal hypertension are primarily mechanical and reversible, without evidence of clinically significant impairment of oxygenation. In contrast, arterial carbon dioxide levels showed no statistically significant changes across experimental phases, indicating preserved ventilatory compensation despite increasing intra-abdominal pressure. Similarly, base excess did not demonstrate statistically significant alterations, suggesting relative preservation of systemic metabolic compensation. Importantly, although PaO₂ and PaO₂/FiO₂ values decreased with increasing intra-abdominal pressure, they largely remained within physiological ranges. These findings are consistent with early physiological alterations rather than overt respiratory dysfunction. Only a small number of animals demonstrated values approaching the lower limits of normal, while the overall mean PaO₂/FiO₂ remained high even at the highest intra-abdominal pressure level.

Arterial carbon dioxide levels remained relatively stable across experimental phases, with only a modest, non-significant increase observed at higher intra-abdominal pressure levels. These findings suggest that, under controlled ventilatory conditions, carbon dioxide elimination was largely preserved despite mechanical respiratory impairment, consistent with numerous reports in the literature (40, 41). Differences in ventilatory models, duration of increased pressure exposure, and experimental design likely explain these discrepancies.

Although arterial pH demonstrated a significant phase-dependent decrease, reflecting mild respiratory and metabolic disturbances, base excess did not show statistically significant changes. This suggests that the observed acid–base alterations were relatively limited and effectively buffered, without evidence of pronounced metabolic derangement. Collectively, these findings indicate that the respiratory effects of intra-abdominal hypertension are primarily driven by impaired oxygenation and mild acid–base shifts, rather than by significant disturbances in carbon dioxide homeostasis.

Hemodynamic changes to elevated IAP were characterized by increased filling pressures and systemic vascular resistance, while cardiac output was preserved through adequate volume support. This observation is consistent with contemporary experimental and clinical data demonstrating that the impact of IAH on cardiac output depends significantly on intravascular volume status (42). When normovolemia is maintained, elevated IAP may coexist with preserved or even increased mean arterial pressure due to compensatory vasoconstriction, despite significant alterations in venous return and preload conditions (43). Preservation of cardiac output in the present experimental setting was intentional, allowing assessment of renal oxygenation independent of global circulatory compromise.

The observed increases in pulmonary artery pressure, pulmonary capillary wedge pressure, and central venous pressure levels correspond to the pressure transmission from the abdominal to the thoracic compartment rather than intrinsic cardiac dysfunction. Previous studies have emphasized that filling pressures measured during intra-abdominal hypertension may overestimate true cardiac preload due to external pressure effects, an important consideration when interpreting hemodynamic data in this setting (44, 45).

An important observation of the present study is the significant reduction in renal cortical tissue oxygenation despite preserved cardiac output and systemic oxygen delivery. This finding provides direct experimental evidence that renal hypoxia during IAH can occur independently of macrocirculatory failure. Recent clinical studies have similarly shown that AKI may occur in patients with IAH even when conventional hemodynamic parameters remain within acceptable ranges (46). The aforementioned findings support the hypothesis that intrarenal microcirculatory disturbances hold a dominant role in the pathogenesis of IAH-associated renal dysfunction.

Although renal perfusion pressure remained above traditionally cited thresholds during the experiment, renal cortical hypoxia developed early and progressed with increasing IAP. This finding indicates that maintenance of renal perfusion pressure alone may be insufficient to preserve renal oxygenation under conditions of elevated IAP. Data drawn both from experimental and clinical studies highlight that venous congestion, increased interstitial pressure, and external parenchymal compression impair renal microvascular flow and promote redistribution of intrarenal blood flow from cortex to medulla, resulting in functional shunting and cortical hypoxia, although this mechanism has not been directly confirmed in the present study (47, 48).

The reduction in renal tissue oxygenation observed in our experiment was not associated with impaired arterial oxygen content, as hemoglobin concentration and oxygen saturation remained stable. This observation is consistent with prior experimental reports demonstrating that renal tissue oxygen tension is relatively insensitive to increases in inspired oxygen fraction and is instead primarily affected by microvascular perfusion and intrarenal blood flow distribution (49–51). These results reflect the limitations of systemic oxygenation parameters as indirect indicators of renal tissue oxygenation.

Urine output declined significantly with increasing IAP and did not fully recover after decompression. Early reductions in diuresis at IAP levels as low as 15–20 mmHg have been repeatedly reported and rank among the earliest clinical manifestations of intra-abdominal hypertension (46, 52). The modest degree of persistent oliguria observed in this study likely reflects the relatively short duration of pressure exposure and preserved renal perfusion pressure. Notably, the primary aim of this experiment was not to reproduce advanced renal failure, but to characterize early oxygenation changes that may precede overt functional deterioration.

The principal strength of this study is that it provides the first focused, direct, continuous assessment of renal cortical oxygenation under controlled conditions of isolated IAH and conditions of preserved cardiac output, oxygen delivery, and arterial oxygenation. In contrast to the study by Kalfadis et al. (28), which examined renal tissue oxygenation in a canine model combining ACS with systemic sepsis, the current trial deliberately excluded any inflammatory and infectious confounders. This reductionist design ensures discrimination between the direct impact of increased IAP and the secondary effects of systemic inflammatory stress.

By minimizing systemic confounders and maintaining stable hemodynamics and oxygen transport variables, this experiment assessed renal cortical tissue hypoxia in the absence of global circulatory compromise, providing powerful evidence that elevated IAP alone is sufficient to develop clinically relevant renal cortical hypoxia. By isolating mechanical pressure as the primary factor, the present study complements earlier work and extends current understanding of the pathophysiological sequence leading to ACS-associated AKI. These findings reinforce the concept of an intrarenal compartment syndrome and highlight the potential value of tissue-level monitoring for early detection of renal failure.

Although changes were observed in respiratory, hemodynamic, and renal parameters, these values largely remained within physiological ranges and therefore do not represent overt organ dysfunction. Instead, these findings likely reflect early, preclinical physiological alterations. In particular, the reduction in renal cortical tissue oxygen tension, in parallel with changes in respiratory mechanics and hemodynamic variables, may indicate the initial stages of organ impairment preceding clinically overt dysfunction. These observations suggest that such parameters could serve as early indicators, enabling timely interventions to prevent progression to established organ failure.

It should be acknowledged that, within the conditions of the present experimental model, overt organ dysfunction at the respiratory, hemodynamic, or renal level was not fully established. Instead, the observed changes in physiological parameters likely reflect an early, preclinical stage of organ impairment. In particular, the reduction in renal cortical tissue oxygen tension, along with alterations in respiratory mechanics and hemodynamic parameters, suggests the presence of early pathophysiological disturbances preceding clinically overt dysfunction. These findings have potential clinical relevance, as early detection of such physiological alterations may allow timely implementation of corrective interventions aimed at preventing progression to established organ failure. Accordingly, the present findings should be interpreted as reflecting early, preclinical alterations rather than established organ dysfunction, highlighting the potential value of continuous physiological monitoring for early detection and prevention strategies.

This study has several limitations that must be acknowledged. It was conducted in an experimental animal model, which may limit direct translation to clinical settings. The sample size was relatively small, and follow-up after decompression was short, precluding evaluation of long-term renal recovery. Moreover, tissue oximetry, although highly informative, is invasive and may introduce minor local trauma that could influence measurements. An additional limitation of the present experimental study is that renal tissue oxygenation was assessed exclusively at the cortical level, without simultaneous measurement of medullary oxygen tension. Consequently, the hypothesis of intrarenal blood flow redistribution from cortex to medulla cannot be directly confirmed in this experimental model and remains based on indirect physiological interpretation and previously published data. Future studies incorporating simultaneous cortical and medullary oxygen measurements would be required to validate this mechanism. In addition, although urine output was continuously measured, urine osmolality was not evaluated. This limits the ability to determine whether the observed reduction in diuresis was associated with increased ADH activity. Measurement of urine osmolality could have provided important insights into the contribution of hormonal regulation to renal dysfunction under conditions of intra-abdominal hypertension. An additional limitation of the present study is the absence of inflammatory, microcirculatory, and tubular injury markers, such danger-associated molecular patterns, circulating interleukins, sidestream dark-field imaging for microcirculatory assessment, and neutrophil gelatinase-associated lipocalin. These parameters could have provided additional insight into early subclinical renal injury. However, the study was deliberately designed as a reductionist model to isolate the direct effects of intra-abdominal pressure on renal oxygenation. Within this context, the observed early decline in renal cortical ptiO₂, in parallel with reduced diuresis despite preserved systemic hemodynamics, supports pressure-induced intrarenal hypoxia as an early event. Future studies incorporating these markers are warranted to further clarify underlying mechanisms. Finally, histological assessment was not performed, which might have provided mechanistic insight through additional data from structural correlates of hypoxia. These limitations notwithstanding, the present findings offer important mechanistic insight into the pathophysiology of renal dysfunction during IAH and lay the groundwork for future experimental and clinical investigations.

Taken together, the observed changes in respiratory, hemodynamic, and metabolic parameters reflect an early, preclinical stage of organ impairment rather than established organ dysfunction. Despite statistically significant alterations in selected variables, most parameters remained within physiological ranges, indicating preserved global function. In contrast, renal cortical oxygen tension and diuresis demonstrated more pronounced and persistent disturbances, highlighting the particular vulnerability of the kidney to intra-abdominal hypertension. These observations support the concept that continuous physiological monitoring may enable early detection of organ stress before the onset of overt clinical failure.

## 5 Conclusions

Our study demonstrates that increasing IAP progressively induces significant respiratory and hemodynamic disturbances, manifested by reductions in arterial oxygen tension, minimal changes in arterial carbon dioxide levels, deterioration of lung compliance, and elevations in intrathoracic and vascular pressures. Importantly, renal tissue oxygenation declined in an inverse relationship with IAP, independent of cardiac output or mean arterial pressure. Supplemental oxygen and fluctuations in arterial PaO₂ did not improve cortical oxygen tension once hemoglobin saturation was near maximal levels.

These findings suggest that during intra-abdominal hypertension, the kidney enters a state of relative hypoxia, potentially mediated, among other mechanisms, by increased antidiuretic hormone release, although this was not directly assessed in the present study, and by intrarenal shunting of blood flow from cortex to medulla due to parenchymal compression. Notably, surgical decompression resulted in substantial recovery of respiratory, hemodynamic, and renal parameters to baseline, underscoring the dynamic and potentially reversible nature of these pathophysiologic changes when intervention is timely.

## Data Availability

The original contributions presented in the study are included in the article/Supplementary Material, further inquiries can be directed to the corresponding author.
