# Supplementary material for: Contribution of tissue oximetry to the assessment of renal dysfunction in abdominal compartment syndrome: an experimental canine study
Source: Front Surg. 2026 May 21;13:1775062. doi: 10.3389/fsurg.2026.1775062 (PMC13233684; doi:10.3389/fsurg.2026.1775062)
Supplement: Supplementary file 1 [file Table1.docx]

|  | Animal No 1 | | | | Animal No 2 | | | |
| --- | --- | --- | --- | --- | --- | --- | --- | --- |
|  | Phase 1  0 mmHg | Phase 2  15 mmHg | Phase 3  30 mmHg | Phase 4  0 mmHg | Phase 1  0 mmHg | Phase 2  15 mmHg | Phase 3  30 mmHg | Phase 4  0 mmHg |
| FiO₂ | 0,4 | 0,4 | 0,4 | 0,4 | 0,4 | 0,4 | 0,4 | 0,4 |
| PaO₂ | 122 | 106 | 101 | 103 | 289 | 303 | 290 | 309 |
| PaO_2_/FiO_2_ | \| 305 \| \| --- \| | 265 | 252.5 | 257.5 | 722.5 | 757.5 | 725 | 772.5 |
| PaCO₂ | 37 | 35 | 41 | 38 | 37 | 35 | 38 | 30 |
| pH | 7,36 | 7,33 | 7,32 | 7,35 | 7,37 | 7,35 | 7,32 | 7,36 |
| Base Excess (BE) | -4 | -7 | -6 | -7 | -6 | -5 | -7 | -4 |
| Hemoglobin Oxygen Saturation (SaO₂) | 100 | 97 | 99 | 99 | 100 | 100 | 99 | 100 |
| Peak Airway Pressure | 12 | 17 | 18 | 11 | 25,8 | 27,5 | 29 | 29 |
| Lung Compliance | 17,4 | 11 | 8,9 | 22 | 20,1 | 17,1 | 17,2 | 17,6 |
| Heart Rate (HR) | 70 | 75 | 75 | 70 | 110 | 127 | 130 | 133 |
| Mean Arterial Pressure (MAP) | 80 | 110 | 95 | 110 | 114 | 126 | 128 | 107 |
| Mean Pulmonary Artery Pressure (MPAP) | 6 | 8 | 7 | 7 | 7 | 8 | 6 | 6 |
| Pulmonary Capillary Wedge Pressure (PCWP) | 2 | 5 | 5 | 3 | 4 | 4 | 5 | 4 |
| Central Venous Pressure (CVP) | 5 | 8 | 9 | 6 | 2 | 2 | 3 | 3 |
| Cardiac Output (CO) | 6,1 | 7 | 5,1 | 7,8 | 4 | 4,7 | 4,6 | 4 |
| Cardiac Index (CI) | 8,5 | 9,7 | 7,1 | 8,3 | 5,6 | 6,5 | 6,4 | 5,5 |
| Oxygen Consumption (VO₂) | 305 | 280 | 204 | 240 | 160 | 188 | 138 | 160 |
| Oxygen Delivery (DO₂) | 793 | 910 | 663 | 780 | 722 | 611 | 598 | 220 |
| Arteriovenous Oxygen Difference [D(a–v)O₂] | 5 | 4 | 4 | 4 | 4 | 4 | 3 | 4 |
| Systemic Vascular Resistance Index (SVRI) | 706 | 841 | 969 | 1002 | 1071 | 1525 | 1562 | 1485 |
| Pulmonary Vascular Resistance Index (PVRI) | 38 | 25 | 23 | 39 | 57 | 49 | 12 | 29 |
| Renal Cortical Tissue Oxygen Tension (ptiO₂) | 250 | 112 | 90 | 144 | 69 | 55 | 49 | 63 |
| Urine Output | 100 | 25 | 35 | 20 | 500 | 300 | 100 | 100 |

|  | Animal No 3 | | | | Animal No 4 | | | |
| --- | --- | --- | --- | --- | --- | --- | --- | --- |
|  | Phase 1  0 mmHg | Phase 2  15 mmHg | Phase 3  30 mmHg | Phase 4  0 mmHg | Phase 1  0 mmHg | Phase 2  15 mmHg | Phase 3  30 mmHg | Phase 4  0 mmHg |
| FiO₂ | 0,4 | 0,4 | 0,4 | 0,4 | 0,4 | 0,4 | 0,4 | 0,4 |
| PaO₂ | 327 | 310 | 287 | 310 | 244 | 166 | 165 | 230 |
| PaO_2_/FiO_2_ | 817.5 | 775 | 717.5 | 775 | 610 | 415 | 412.5 | 575 |
| PaCO₂ | 35 | 36 | 36 | 35 | 46 | 48 | 47 | 45 |
| pH | 7,47 | 7,42 | 7,46 | 7,43 | 7,35 | 7,35 | 7,35 | 7,36 |
| Base Excess (BE) | 2,4 | 0 | 2 | 1 | 0,4 | 0 | 0 | 0 |
| Hemoglobin Oxygen Saturation (SaO₂) | 100 | 100 | 100 | 100 | 99 | 99 | 99,5 | 100 |
| Peak Airway Pressure | 14 | 19 | 21 | 13 | 18 | 22 | 23 | 17 |
| Lung Compliance | 15 | 12 | 9 | 13 | 20 | 19 | 19 | 20 |
| Heart Rate (HR) | 75 | 77 | 80 | 73 | 78 | 85 | 89 | 75 |
| Mean Arterial Pressure (MAP) | 63 | 71 | 69 | 65 | 68 | 81 | 85 | 66 |
| Mean Pulmonary Artery Pressure (MPAP) | 8 | 9 | 9 | 8 | 9 | 10 | 11 | 8 |
| Pulmonary Capillary Wedge Pressure (PCWP) | 3 | 6 | 6 | 4 | 4 | 4 | 5 | 4 |
| Central Venous Pressure (CVP) | 2 | 4 | 3 | 3 | 3 | 3 | 4 | 3 |
| Cardiac Output (CO) | 3,8 | 3,9 | 4,1 | 4,2 | 5,2 | 5 | 5,1 | 4,9 |
| Cardiac Index (CI) | 5,3 | 4,9 | 5,7 | 5,8 | 7,2 | 6,9 | 7,1 | 6,8 |
| Oxygen Consumption (VO₂) | 152 | 156 | 164 | 168 | 208 | 200 | 204 | 196 |
| Oxygen Delivery (DO₂) | 686 | 507 | 533 | 546 | 676 | 650 | 663 | 637 |
| Arteriovenous Oxygen Difference [D(a–v)O₂] | 4 | 4 | 4 | 4 | 4 | 4 | 4 | 4 |
| Systemic Vascular Resistance Index (SVRI) | 920 | 1093 | 926 | 855 | 722 | 904 | 732 | 741 |
| Pulmonary Vascular Resistance Index (PVRI) | 75 | 49 | 42 | 55 | 56 | 70 | 35 | 47 |
| Renal Cortical Tissue Oxygen Tension (ptiO₂) | 220 | 119 | 90 | 101 | 185 | 153 | 114 | 148 |
| Urine Output | 200 | 180 | 160 | 160 | 220 | 200 | 200 | 210 |

|  | Animal No 5 | | | | Animal No 6 | | | |
| --- | --- | --- | --- | --- | --- | --- | --- | --- |
|  | Phase 1  0 mmHg | Phase 2  15 mmHg | Phase 3  30 mmHg | Phase 4  0 mmHg | Phase 1  0 mmHg | Phase 2  15 mmHg | Phase 3  30 mmHg | Phase 4  0 mmHg |
| FiO₂ | 0,4 | 0,4 | 0,4 | 0,4 | 0,4 | 0,4 | 0,4 | 0,4 |
| PaO₂ | 360 | 290 | 260 | 350 | 239 | 195 | 190 | 205 |
| PaO_2_/FiO_2_ | 900 | 725 | 650 | 875 | 597.5 | 487.5 | 475 | 512.5 |
| PaCO₂ | 31 | 35,3 | 26 | 32 | 29 | 30 | 31 | 30 |
| pH | 7,48 | 7,41 | 7,41 | 7,47 | 7,48 | 7,47 | 7,46 | 7,47 |
| Base Excess (BE) | 0,7 | -1,5 | -1 | 0 | -0,2 | -0,1 | 0 | 0 |
| Hemoglobin Oxygen Saturation (SaO₂) | 100 | 100 | 100 | 100 | 100 | 100 | 100 | 100 |
| Peak Airway Pressure | 10 | 14 | 17 | 12 | 21 | 24 | 27 | 23 |
| Lung Compliance | 16 | 10 | 10 | 15 | 18 | 15 | 14 | 19 |
| Heart Rate (HR) | 84 | 86 | 83 | 85 | 95 | 98 | 99 | 90 |
| Mean Arterial Pressure (MAP) | 65 | 73 | 77 | 71 | 84 | 93 | 97 | 90 |
| Mean Pulmonary Artery Pressure (MPAP) | 6 | 8 | 7 | 5 | 7 | 7 | 8 | 7 |
| Pulmonary Capillary Wedge Pressure (PCWP) | 2 | 2 | 3 | 2 | 4 | 4 | 5 | 4 |
| Central Venous Pressure (CVP) | 2 | 3 | 3 | 2 | 3 | 3 | 3 | 2 |
| Cardiac Output (CO) | 3,2 | 3,4 | 3,3 | 3,5 | 4,3 | 4,5 | 4,8 | 4,4 |
| Cardiac Index (CI) | 4,4 | 4,7 | 4,6 | 4,9 | 6 | 6,2 | 6,7 | 6,1 |
| Oxygen Consumption (VO₂) | 128 | 200 | 132 | 140 | 559 | 136 | 240 | 176 |
| Oxygen Delivery (DO₂) | 578 | 422 | 429 | 455 | 215 | 442 | 624 | 572 |
| Arteriovenous Oxygen Difference [D(a–v)O₂] | 4 | 4 | 4 | 4 | 5 | 5 | 5 | 4 |
| Systemic Vascular Resistance Index (SVRI) | 1145 | 1191 | 1246 | 1126 | 1079 | 1161 | 1122 | 1154 |
| Pulmonary Vascular Resistance Index (PVRI) | 73 | 102 | 73 | 49 | 40 | 39 | 36 | 39 |
| Renal Cortical Tissue Oxygen Tension (ptiO₂) | 215 | 212 | 102 | 117 | 196 | 116 | 98 | 132 |
| Urine Output | 300 | 250 | 250 | 280 | 150 | 130 | 130 | 140 |

|  | Animal No 7 | | | | Animal No 8 | | | |
| --- | --- | --- | --- | --- | --- | --- | --- | --- |
|  | Phase 1  0 mmHg | Phase 2  15 mmHg | Phase 3  30 mmHg | Phase 4  0 mmHg | Phase 1  0 mmHg | Phase 2  15 mmHg | Phase 3  30 mmHg | Phase 4  0 mmHg |
| FiO₂ | 0,4 | 0,4 | 0,4 | 0,4 | 0,4 | 0,4 | 0,4 | 0,4 |
| PaO₂ | 237 | 170 | 163 | 244 | 110 | 107 | 100 | 103 |
| PaO_2_/FiO_2_ | 592.5 | 425 | 407.5 | 610 | 275 | 267.5 | 250 | 257.5 |
| PaCO₂ | 46,5 | 47,7 | 48 | 46,4 | 34 | 38 | 38 | 35 |
| pH | 7,35 | 7,34 | 7,34 | 7,35 | 7,47 | 7,46 | 7,46 | 7,46 |
| Base Excess (BE) | -0,3 | -0,2 | -0,3 | 0,4 | 2 | 1,8 | 1,7 | 2 |
| Hemoglobin Oxygen Saturation (SaO₂) | 100 | 100 | 100 | 100 | 100 | 100 | 100 | 100 |
| Peak Airway Pressure | 12,5 | 15,5 | 14 | 11 | 21 | 24,5 | 25 | 22 |
| Lung Compliance | 19 | 18 | 15 | 20 | 19,5 | 19 | 19,3 | 19 |
| Heart Rate (HR) | 107 | 113 | 110 | 105 | 69 | 73 | 75 | 70 |
| Mean Arterial Pressure (MAP) | 84 | 88 | 90 | 80 | 74 | 79 | 78 | 81 |
| Mean Pulmonary Artery Pressure (MPAP) | 9 | 10 | 9 | 9 | 7 | 8 | 8 | 8 |
| Pulmonary Capillary Wedge Pressure (PCWP) | 3 | 3 | 3 | 3 | 5 | 6 | 7 | 6 |
| Central Venous Pressure (CVP) | 3 | 4 | 3 | 3 | 3 | 4 | 6 | 3 |
| Cardiac Output (CO) | 5,2 | 5 | 5,1 | 5,3 | 5,8 | 5,7 | 5,3 | 5,5 |
| Cardiac Index (CI) | 7,2 | 6,9 | 7,1 | 7,4 | 8,1 | 7,9 | 7,4 | 7,6 |
| Oxygen Consumption (VO₂) | 208 | 200 | 204 | 212 | 232 | 228 | 212 | 220 |
| Oxygen Delivery (DO₂) | 676 | 650 | 663 | 689 | 754 | 741 | 689 | 715 |
| Arteriovenous Oxygen Difference [D(a–v)O₂] | 4 | 4 | 4 | 4 | 4 | 4 | 4 | 4 |
| Systemic Vascular Resistance Index (SVRI) | 911 | 973 | 980 | 832 | 701 | 759 | 778 | 810 |
| Pulmonary Vascular Resistance Index (PVRI) | 44 | 81 | 68 | 65 | 20 | 20 | 11 | 21 |
| Renal Cortical Tissue Oxygen Tension (ptiO₂) | 87 | 64 | 58 | 71 | 163 | 135 | 102 | 147 |
| Urine Output | 160 | 160 | 150 | 170 | 240 | 200 | 190 | 200 |

| **Parameter** | **Unit** |
| --- | --- |
| FiO₂ | fraction (–) |
| PaO₂ | mmHg |
| PaCO₂ | mmHg |
| pH | – |
| Base Excess (BE) | mmol/L |
| Hemoglobin Oxygen Saturation (SaO₂) | % |
| Peak Airway Pressure | cmH₂O |
| Lung Compliance | mL/cmH₂O |
| Heart Rate (HR) | beats/min |
| Mean Arterial Pressure (MAP) | mmHg |
| Mean Pulmonary Artery Pressure (MPAP) | mmHg |
| Pulmonary Capillary Wedge Pressure (PCWP) | mmHg |
| Central Venous Pressure (CVP) | mmHg |
| Cardiac Output (CO) | L/min |
| Cardiac Index (CI) | L/min/m² |
| Oxygen Consumption (VO₂) | mL/min |
| Oxygen Delivery (DO₂) | mL/min |
| Arteriovenous Oxygen Difference [D(a–v)O₂] | mL O₂/dL blood |
| Systemic Vascular Resistance Index (SVRI) | dyn·s·cm⁻⁵·m² |
| Pulmonary Vascular Resistance Index (PVRI) | dyn·s·cm⁻⁵·m² |
| Renal Cortical Tissue Oxygen Tension (ptiO₂) | mmHg |
| Urine Output | mL/30 min |
